# Chorea

**Published:** 1874-11

**Authors:** 


The Treatment of Chorea.—Dr. Anstie, at the conclusion of a series of admirable papers (Practitioner) on chorea, says: * * *

"In the excessively severe case of the boy, we tried not only conium, but bromide of potassium, camphor, ol. morrhuæ, and zinc in large doses, all with only momentary effect. We then tried Jaccoud's plan, which I have found extraordinarily successful in several cases, viz: we sprayed the skin over the whole length of the spine with ether twice daily. I will not positively say that it was *propter hoc*, but I will say that immediately *post hoc* the symptoms greatly amended, and in the course of a fortnight the lad was perfectly free from movements. The girl, with whom succus conii, camphor, ol. morrhuæ, bromide of potassium, and large doses of zinc had entirely failed, began to improve immediately on taking liq. arsenicalis in five-minim doses, afterwards reduced to three minims. I am convinced that in one of these cases death, and in the other a protracted and very serious illness were avoided by the use of remedies; and I will just say here that arsenic as an internal remedy, and the ether spray applied to the spine, have given me solid results, such as have been obtained by no other remedy. The ether spray stands somewhat intermediate, I suppose, between the ordinary shower-bath and the spinal ice-bags, of which so much has been said. Cod-liver oil and iron, however, are very useful in anæmic and generally debilitated subjects. And there is a special class of cases connected with violent ovarian excitement, or complicated with epileptic tendencies, in which the bromide of potassium is invaluable, and is the one remedy.

"In the terribly dangerous acute cases of young women, especially where there has been sexual excitement and exhaustion, I believe nothing does any good but free stimulation, regular feeding per rectum, and opium in large doses. I regret to have to express my complete distrust in chloral and in a host of other remedies that have been proposed.—*American Practitioner.*

